# Camouflage patterning modulates neural signatures of attention and decision-making

**DOI:** 10.1098/rspb.2024.0865

**Published:** 2024-08-14

**Authors:** Jac Billington, Christopher Hassall, Matt Craddock

**Affiliations:** ^1^ School of Psychology, Faculty of Medicine and Health, University of Leeds, Leeds, UK; ^2^ School of Biology, Faculty of Biological Sciences, University of Leeds, Leeds, UK; ^3^ Alan Turing Institute, London, UK

**Keywords:** camouflage, attention, decision-making, neural, EEG

## Abstract

Many animals rely on visual camouflage to avoid detection and increase their chances of survival. Edge disruption is commonly seen in the natural world, with animals evolving high-contrast markings that are incongruent with their real body outline in order to avoid recognition. While many studies have investigated how camouflage properties influence viewer performance and eye movement in predation search tasks, researchers in the field have yet to consider how camouflage may directly modulate visual attention and object processing. To examine how disruptive coloration modulates attention, we use a visual object recognition model to quantify object saliency. We determine if object saliency is predictive of human behavioural performance and subjective certainty, as well as neural signatures of attention and decision-making. We show that increasing edge disruption not only reduces detection and identification performance but is also associated with a dampening of neurophysiological signatures of attentional filtering. Increased self-reported certainty regarding decisions corresponds with neurophysiological signatures of evidence accumulation and decision-making. In summary, we have demonstrated a potential mechanism by which edge disruption increases the evolutionary fitness of animals by reducing the brain’s ability to distinguish signal from noise, and hence to detect and identify the camouflaged animal.

## 1. Introduction

Over the past century, ecologists have made strides in studying the fundamental principles behind animal signalling [1]. Among this body of work, several traits have been defined as ways that species can avoid being detected [1]. Visual camouflage is a common means of avoiding detection, including background matching (e.g. blending into the background because of matched colour, contrast, texture frequency [2]), disruptive camouflage (e.g. the use of visual patterning to prevent easy recognition of prominent features [3,4]) and self-shadow concealment via countershading to enhance survival probability [5,6]. Edge disruption is a particular aspect of disruptive camouflage in which high-contrast patterns are aligned with the edge of an animal’s body [7]. Edge disruption prevents or slows the detection and recognition of the animal in visual search paradigms with artificial prey [4] and modulates known observable eye movements in eye-tracking experiments that are considered signatures of viewer attention [8]. For example, artificial prey with more intersecting edge disruption patches are passed over more during visual search (i.e. gaze lands on the prey but does not linger, and is fixated on for longer periods of time prior to active recognition and capture or decision to attack [4]).

While eye-tracking can inform us about overt visual attention to camouflaged targets [4,8], we do not yet understand how camouflaged markings affect the initial mechanisms that guide attention. During ‘covert’ deployments of attention an object can be detected, and a decision is made regarding the object’s properties without, or before, any observable eye movement [9]. Thus, previous methods of measuring how camouflage affects search performance may miss informative data due to their limited measurement capabilities. To be rigorous in our understanding of the impact of camouflaged markings on both attention and object processing, we need the ability to measure internal cognitive processes and achieve finer temporal resolution in data sampling. This can be accomplished using electroencephalogram (EEG) technology, which records event-related potentials (ERPs), which are distinct neural signatures time-locked to a stimulus onset or participant response. As such, ERP signatures are capable of tracking early covert reallocations of attention [10] and can be used to understand how variations in animal patterning modulate specific attentional and decision-making processes.

Recognizing a camouflaged object involves two processes: (i) identifying that there may be an object at a particular location and (ii) demarcating its edges to determine its appearance or shape. These processes are associated with known ERP signatures. The N2pc (negative 200 posterior contralateral) occurs when a stimulus prompts a shift in attention [11], such as might occur when a prey target enters the field of vision. It occurs during early covert attentional processes, meaning it can happen before (or even without) overt eye movements towards the stimulus that has captured attention [12]. The N2pc manifests in response to abrupt changes and discontinuities in the visual environment, such as an object against a background [13] or where distracting sensory inputs, irrelevant to the task at hand, need to be disregarded to focus attention on and respond to a pertinent target [14]. As such, we might expect that prey that stand out against their background would trigger a strong N2pc response. The N2pc is believed to reflect two primary processes. Firstly, it is thought to represent a discriminative filtering process, responsible for suppressing non-relevant information in the visual scene while heightening attention to relevant information [14]. Secondly, more recent research has shown that it also reflects the process of converting raw visual sensory input into a coherent neural representation, aiding object identification [9,15]. In naturalistic settings, failure to detect a camouflaged object in the visual scene could plausibly indicate a breakdown in one or both processes: camouflaged prey are either discarded as background ‘noise’ or do not match search images during recognition.

As the process of attending to and identifying an object unfolds, a sustained posterior contralateral negativity (SPCN) is often observed [16]. The SPCN is commonly associated with the sustained maintenance of visuospatial attention, and it is observed in tasks where participants are required to remember and/or attend to a specific location or feature in the visual field over a period of time, such as the course of an experimental trial [16]. The SPCN is thought to reflect holding and updating an object representation in short-term working memory in the lead up to the decision response, even if the task is not designed as an explicit memory task *per se* [16,17]. During a predator–prey encounter, a camouflaged prey target would potentially elicit a weaker SPCN, as the predator would remain searching for targets rather than focusing on a specific location where a target had been detected.

Studies on decision-making based on perceptual information have identified an ERP known as central parietal positivity (CPP) [18]. This signal tends to build in amplitude over time as individuals gather more sensory information, a phenomenon often referred to as evidence accumulation [18]. The CPP is readily observed in experimental paradigms where participants are required to continuously monitor a stimulus that gradually changes over time and make a response decision when a particular property reaches a certain threshold [18]. The CPP is also modulated by the self-reported subjective certainty in the decision response [19]. This suggests that CPP may also represent the ‘conscious experience’ during the evidence accumulation process, rather than solely reflecting the physical stimulus characteristics. Variation in subjective certainty on a trial-by-trial basis, even with the same physical stimulus, is thought to result from internal neural noise influencing the evidence accumulation process, which is subsequently reflected in the participant’s decision response [19]. Thus, both the strength of the signal (i.e. physical stimulus properties) and the level of noise (i.e. inherent neural noise) are important factors that lead to a decision. This theory accounts for erroneous responses in experiments requiring stimulus detection (i.e. false positives and false negatives). The discovery that observers can spend a relatively extended amount of time looking directly at a camouflaged stimulus before deciding to make an attack [4] may reflect the role of camouflage in exploiting this signal-to-noise balance and, thus, slowing the evidence accumulation and decision-making process. Hence, via CPP, a camouflaged prey target may slow object recognition and create a time lag in the associated attack behaviour by the predator. This could occur in addition to, or indeed, instead of, camouflage influencing the attentional spotlight of the observer in the first place.

In this study, we focus on these three ERP signatures to determine whether edge disruption elicits discernible differences in attention, object recognition and decision-making in a detection and discrimination task. We modify a traditional attentional cueing paradigm [20] by presenting a camouflaged cue, which enables us to assess how cues with varying levels of edge disruption redirect attention to a forthcoming target. We measure reaction times (RT), accuracy rates and subjective decision certainty, alongside the ERP signatures. We hypothesized that as the degree of edge disruption increased, both the N2pc and SPCN components would decrease in amplitude, indicating a decline in the effectiveness of attentional allocation, and a reduction in the efficiency of maintaining the neural representation of the object. We also hypothesized that heightened edge disruption would correlate with a reduction in the amplitude of the CPP due to impoverished evidence accumulation. We predicted that the relationship between levels of edge disruption and neural signatures may be mediated by self-reported subjective certainty, reflecting a decline in the neural signal-to-noise ratio in the evidence accumulation processes during detection of harder to detect stimulus.

## 2. Methods

### (a) Participants

Twenty-one adult participants were recruited from the local community via e-mails sent to a departmental participant database. Two participants were excluded from the analysis: one was unable to complete the task due to difficulty in seeing the camouflaged stimuli and another had no recorded behavioural data due to equipment failure. All participants were screened for corrected-to-normal vision and the absence of neurological conditions. The remaining participants included 9 males and 10 females, with a mean age of 32.1 years (s.d. = 13.1, range: 19–60).

### (b) EEG task

**Figure 1.**
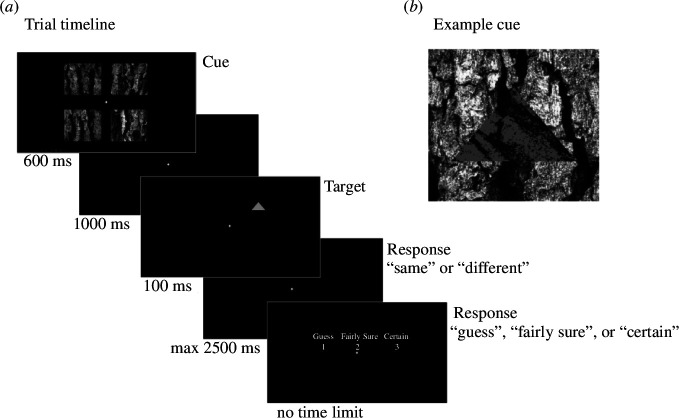
Trial design and stimulus properties. (*a*) Trial sequence (the cue in panel 1 is located in the top right corner). (*b*) Example cue stimulus.

We used a cuing paradigm (figure 1*a*) in which two stimuli were presented in sequence within a trial (referred to as *cue* and *target* hereafter). A total of 348 trials per participant were presented during the EEG study, utilizing Psychtoolbox for stimulus presentation [21]. Following an initial crosshair presentation for 600 ms, a camouflaged cue stimulus was shown on one of four bark backgrounds for 600 ms. The stimuli were presented centrally within a visual angle of 23.1 × 22.0°, with each individual bark patch presented in a square configuration at a visual angle of 10.0 × 9.0°. The cue stimuli comprised a tree bark textured triangle presented on a tree bark textured background (figure 1*b*). The cue and backgrounds were created from the same image of an English oak, *Quercus robur*, and all bark patches were luminance matched within each trial to avoid confounding influences on attention (see electronic supplementary material, S3 for further details regarding stimulus creation). The target was a uniform grey colour (50 percentile of grey scale of the original bark image) and presented for 100 ms following an interim 1000 ms crosshair presentation (±50 ms jitter).

The cue stimuli varied in the level of edge disruption (EdgeDis) as a result of random sampling across the bark surface. The degree of EdgeDis was quantified as a ratio of orthogonal to parallel orientations within edge zones, computationally determined [22] using a visual object recognition model [6,23] (see electronic supplementary material, S2). Studies on EdgeDis and background matching have used a similar approach, employing a GabRat metric [7]. Stimuli also varied randomly in bark texture rotation (TexRot) compared with bark texture background. TexRot could either be 0° (bark texture matched with background bark texture orientation) or 45° (orthogonal texture to background) or 90° (perpendicular texture to background). TexRot was varied to introduce variation in the background matching of the stimulus based on the assumption that the linear striations in the bark pattern would result in increased camouflage if cue stimuli were only extracted from that same orientation (0°). By quantifying this variation, we could model it explicitly, thus avoiding potential confounding effects of local texture matching variations in our naturalistic stimuli. All participants received a continuous range of EdgeDis stimuli and three types of TexRot stimulus. For a detailed discussion, see electronic supplementary material, S3.

Cue and target triangles were presented with an equal probability of the triangle apex pointing up or down for the purpose of the task. To determine if participants had recognized the shape (i.e. discerned orientation) of the triangle cues, they were asked to indicate whether the two triangles presented in sequence had the same orientation (i.e. both with vertex up) or different orientations (i.e. one vertex up and one down). They were given 2500 ms to respond following the presentation of the target, after which a null-response was recorded if no answer was given. After a response (or 2500 ms timeout) participants were asked to give a subjective indication of how sure they were of their response as either guess (0% certainty), fairly sure (more than 0% but not 100%) and certain (100% certain of the answer). Participants were told that fairly sure meant that they did not guess, but they were also not 100% certain. They were not given a restricted time to make this response but were asked not to think too much about this answer. Responses were collected using a Ducky mechanical keyboard with a sampling rate of 1000 Hz (DuckyChannel International, Taiwan). The next trial continued once they had made this response. This delay provided some variation in the inter-stimulus interval itself, helping to prevent response anticipation and reduce EEG artefacts. Trials were presented over five blocks, with self-paced rests in between each block.

The cue presentation screen was designed to emulate a visual search paradigm, wherein the cue could potentially appear in one of four locations. This approach is more controlled compared with the free search method used in many previous studies [4,7]. However, it allowed for the maintenance of gaze fixation throughout the experiment, thereby mitigating the elicitation of eye movement related EEG artefacts resulting from the task itself. Furthermore, the restricted nature of our cue visual search (limited to four locations) would reduce the variability in RT that can result from randomness in the search path trajectory, rather than solely from the attention-capturing properties of the stimuli themselves [24]. By presenting two stimuli in sequence and only requiring a motor response following the second stimulus, attentional ERP responses to the cue could be more reliably dissociated from ERP responses associated with action initiation [23] and decision-making.

In cuing paradigms such as a Posner task [20], the cue would usually reference the correct location of the target on approximately 85% of trials, and incorrectly reference the target on approximately 15% of trials. The effect of attentional cuing can then be measured by comparing responses between the correctly and incorrectly referenced targets. We also took a similar approach in this experiment and the location of the cue correctly referenced the location of the target on approximately 85% of trials (*valid cue*). In the remaining approximately 15% of trials, the target was presented in the opposite visual field but at the same horizontal plane as the cue (*invalid cue*). The heightened probability of the target being in the same position as the cue means that participants should covertly attend to the target location in advance of the target appearing. However, this will only happen if they see the cue clearly and, thus, we can determine how well the camouflage cues reorientated attention and if this response was weaker in trials with higher EdgeDis cues. With invalid trials and high EdgeDis cues, the lower detection rates will lead to reduced attention to the target and so a weaker effect of the invalid cue. With invalid trials and low EdgeDis cues, we would expect a higher detection of the cue and so attention will be more strongly reoriented to the wrong location.

### (c) ERP data processing

EEG data were continuously recorded using a BioSemi ActiveTwo EEG system (BioSemi, Amsterdam, The Netherlands) equipped with 64 electrodes at a sampling rate of 1024 Hz. Initially, the data were down sampled to 512 Hz during preprocessing. Eye movements were concurrently monitored using electro-oculogram (EOG) electrodes positioned lateral to each eye and one above and below the left eye. Additionally, two reference electrodes were affixed to the mastoid bone.

The EEG recording session spanned approximately 30 min, inclusive of self-paced rest breaks. Subsequently, all data preprocessing procedures were performed using EEGlab [25] and Fieldtrip [26] on a Matlab 2019b [27] platform (see electronic supplementary material, S1 for package details). Channels exhibiting exceptionally poor quality, discernible during recording, were excluded. These exclusions amounted to the removal of one channel for four participants and two channels for one participant, all of which were occipital. The continuous data were then re-referenced to the average of the remaining 64 channels. Using EEGlab, a high-pass finite impulse response (FIR) filter was applied to the data using the Kaiser window method [28] with a 1 Hz cut-off, which was utilized to identify artefact components through independent component analysis (ICA). A 0.1 Hz cut-off was subsequently applied for ERP statistical analysis. The extended infomax algorithm [29] was used to identify eye movement and other artefact components in the 1 Hz cut-off dataset. These identified components were used to remove artefacts from the 0.1 Hz cut-off datasets. The datasets with the 0.1 Hz cut-off were processed by dividing them into epochs, ranging from −0.6 to 5.9 s for cue-locked epochs and −1.5 to 0.2 s for response-locked epochs. The fully automated statistical thresholding for EEG artefact rejection (FASTER) toolbox [30] was used for the detection and interpolation of bad channels, as well as the removal of trials with residual noise artefacts. Finally, the processed data were exported to FieldTrip for subsequent statistical analysis. One participant was excluded at the preprocessing stage due to a high number of exceptionally noisy channels.

We employed *a priori* regions of interest (ROI) for our statistical analysis. Specifically, for occipital ERPs, left (O1, O2, PO3) and right (PO4, PO7, PO8) electrodes were selected to compute average waveforms for each hemisphere. In identifying the N2pc, we initially defined the N2 component as the negative deflection occurring between 300 and 400 ms, following clearly identified early visual components P1, N1 and P2. It is worth noting that this deflection occurred slightly later than reported in some studies. We speculate that this delay may be attributed to the reduced distinguishability of the cue from the background. Subsequently, we defined the N2pc as the contralateral N2 (N2c) relative to the ipsilateral N2 (N2i) waveform.

The CPP was derived from the average of electrodes CPZ and Pz. In paradigms requiring manual responses, CPP typically exhibits a characteristic rise, peaking at the time of response. Consequently, we assessed the rise in CPP relative to both the stimulus (i.e. the cue) and the response. For average CPP calculation, we utilized the response-locked time point, ±100 ms. It is important to note that CPP shares many characteristics with the classical P300 component [18,31]. However, while the P300 is conventionally analysed in terms of stimulus-locked ERPs [18,31], our focus here is on response-locked CPP. Therefore, we employ the term CPP to specifically refer to this ERP.

### (d) Statistical analysis

Behavioural data analysis was restricted to trials that were also included in the EEG results presentation. This entailed excluding trials with noisy or excluded EEG data. Following this filtering process, data from 5767 trials across all participants remained, resulting in an average of approximately 27 trials removed per participant. Prior to analysis, behavioural data underwent preprocessing, which involved removing RTs less than 100 ms under the assumption that such responses were too rapid to be valid. Only trials with correct responses were used during RT analysis. Throughout, EdgeDis was treated as a continuous variable, while TexRot was considered a categorical variable with three levels (0°, 45° and 90°).

The statistical analysis of both behavioural data and ERP ROI data was conducted using R packages ‘LmerTest’ for linear and general linear mixed effects models (LMM and GLMM) [32] and ‘nnet’ for multinomial logit models (MLM) [33]. In each case, we include cue validity (CueV), EdgeDis and TexRot as main effects along with the interaction between EdgeDis and TextRot. All models included participant ID as a random effect. Mediation analysis was carried out using the mediation toolbox in R [34]. All model R code and output are available open source, as indexed in electronic supplementary material, S1. A time-series analysis was used to determine if there was an SPCN across the trial. Statistical significance was tested across the time series starting from stimulus onset (0 ms), with a cluster-level thresholding procedure to correct for multiple comparisons [35]. Further statistical analysis details are provided in electronic supplementary material, S4.

## 3. Results

### (a) Edge disruption as a predictor of behavioural performance

We first assessed the behavioural results to compare them with previous studies that have reported increases in camouflaged patterning result in decreased search performance. Overall, accuracy on the task was 77.3%, with 15.5% of responses being incorrect. The remaining 7.3% of trials elicited no response and were coded as incorrect for the purpose of statistical analysis, as these were interpreted as not having seen the cue stimulus. For cue valid trials, accuracy was 78.7%, with an average RT of 952 ms (standard deviation (s.d.) = 391) for correct responses and 1205 ms (s.d. = 536) for incorrect responses. Accuracy for cue invalid trials was 69.4%, with an average RT of 1027 ms (s.d. = 398) for correct responses and 1165 ms (s.d. = 467) for incorrect responses.

CueV emerged as the most significant predictor of RT (χ²(1) = 53.026, *p *< 0.001), with slope estimates indicating faster RT for valid cues, aligning with expectations from a standard cueing paradigm (CueV: *b* = 82.754, standard error (s.e.) = 11.364, confidence intervals (CI) [60.480–105.027]). Additionally, there was an interactive effect of EdgeDis × TexRot (χ²(2) = 17.015, *p *< 0.001), with negative estimates suggesting that the increase in RT with increasing EdgeDis was attenuated in non-0° TexRot stimuli compared with 0° TexRot, and this reduction was more pronounced for 90° (EdgeDis × TexRot.45: *b* = −11.580, s.e. = 9.261, CI [−29.731–6.571]; EdgeDis × TexRot.90: *b* = −44.110, s.e. = 10.927, CI [−65.527 to −22.693]). The main effect of TexRot also predicted RT (χ²(1) = 8.669, *p *< 0.05), while the main effect of EdgeDis on RT was non-significant (χ² (1) = 0.173, *p* = 0.677).

GLMMs were also used to predict accuracy from stimulus characteristics. CueV (χ²(1) = 34.683, *p *< 0.001) and EdgeDis (χ² (1) = 91.861, *p *< 0.001) emerged as predictors of accuracy, with odds ratios (OR) indicating that both an invalid cue and increasing EdgeDis led to lower accuracy (CueV: OR = 0.606, s.e. = 0.052, CI [0.513–0.716]; EdgeDis: OR = 0.713, s.e. = 0.033, CI [0.651–0.780]). Neither the EdgeDis × TexRot interaction (χ²(2) = 5.270, *p* = 0.072) nor the main effect of TexRot (χ² (2) = 2.114, *p* = 0.348) was significant in predicting accuracy.

We explored whether camouflage properties influence the subjective certainty of participants. Overall, 23.5% of trials were reported as guess responses, 26.8% as fairly sure and 49.7% as certain. Accordingly, accuracy within these categories of certainty was 45.2, 75.2 and 94.1%, respectively. A significant main effect of CueV emerged (χ² (2) = 19.514, *p *< 0.001), with estimates indicating that in trials with valid cues, participants were more likely to report higher levels of certainty (fairly sure: *b* = −0.170, s.e. = 0.099, CI [−0.365–0.025]; certain: *b* = −0.397, s.e. = 0.091, CI [−0.576 to −0.218]). Additionally, there was an EdgeDis × TexRot interaction (χ² (4) = 10.175, *p *< 0.05) and a main effect of EdgeDis (χ² (2) = 223.040, *p*<0.001). Although there was an overall decrease in certainty with an increasing amount of EdgeDis (fairly sure: *b* = −0.168, s.e. = 0.120, CI [−0.403–0.067]; certain: *b* = −0.535, s.e. = 0.106, CI [−0.742 to −0.327]), this effect was slightly less pronounced for non-0° TexRot stimuli (EdgeDis × TexRot.45: fairly sure: *b* = −0.172, s.e. = 0.129, CI [−0.425–0.082]; certain: *b* = −0.031, s.e. = 0.115, CI [−0.204–0.386]; EdgeDis × TexRot.90: fairly sure: *b* = 0.091, s.e. = 0.150, CI [−0.256–0.194]; certain: *b* = 0.223, s.e. = 0.135, CI [−0.041–0.486]), reflecting the RT results. No significant main effect of TexRot was observed (χ² (2) = 3.578, *p* = 0.466). Thus, in addition to influencing performance ability on the task, increasing EdgeDis reduced the participants’ subjective self-reported perception of their task performance, particularly when the cue orientation matched the background from which it was extracted.

### (b) Edge disruption as a driver of N2pc

**Figure 2.**
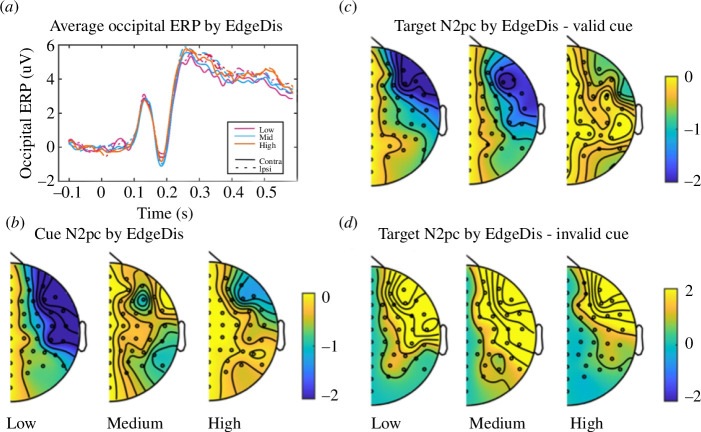
N2pc responses to the cue and target as predicted by EdgeDis. (*a*) Ipsilateral and contralateral occipital ERP responses for low, medium and high EdgeDis stimuli. Stimulus onset and offset were at 0 and 0.6 s, respectively. (*b*) Topographical plots of Cue N2pc (N2c and N2i) for high, medium and low EdgeDis stimuli. (*c*,*d*). Topographical plots of Target N2pc for valid (*c*) and invalid (*d*) cue trials for high, medium and low EdgeDis stimuli. EdgeDis categorization is for display purposes only.

Figure 2*a* illustrates the development of the contralateral and ipsilateral occipital ERP waveforms, including N2, following the cue onset for low, medium and high EdgeDis values over time. The scalp distribution of N2pc at 300–400 ms post-cue onset is displayed in figure 2*b* for low, medium and high EdgeDis values (see electronic supplementary material, figure S5a,b for average N2pc peak values). In predicting the N2pc response immediately following the cue presentation (N2pcCue), there was no significant interaction between EdgeDis and TexRot (F_2,5751_ = 0.509, *p* = 0.601), nor was there a main effect of TexRot (F_2,5750_ = 0.224, *p* = 0.799). However, EdgeDis significantly predicted the N2pcCue (F_1,5749_ = 4.136, *p *< 0.05, *b* = 0.049, s.e. = 0.041, CI [−0.030 to −0.129]). Slope estimates indicate that the N2pcCue response was larger in trials with lower EdgeDis cues, suggesting that lower EdgeDis evoked heightened attentional processing. There was a significant interaction between EdgeDis and CueV on N2pcTarget (F_1,5759_ = 5.762, *p *< 0.05; *b* = 0.007, s.e. = 0.039, CI [−0.172 to −0.017]), and there was a main effect of EdgeDis (F_1,5750_ = 4.101, *p *< 0.05; *b* = 0.095, s.e. = 0.014, CI [−0.020–0.035]), but no main effect of CueV (F_1,5750_ = 1.433, *p* = 0.231). N2pcTarget was greater in low EdgeDis trials when the target location was congruent with the cue location, and vice versa for invalid cues (see figure 2*c*,*d* and electronic supplementary material, figure S5b). Overall, these findings suggest that higher levels of EdgeDis can successfully weaken the reallocation of attention to a forthcoming target location.

### (c) Subjective rating as a mediator of ERP signatures of attention

To explore the relationship between stimulus characteristics, subjective certainty ratings and ERP responses, we conducted a mediation analysis. This analysis focused on the ongoing waveform difference between contralateral and ipsilateral occipital responses (referred to as Npc) to ascertain if an SPCN was modulated by certainty. We also evaluated whether certainty influenced the development of the N2pcCue component.

**Figure 3.**
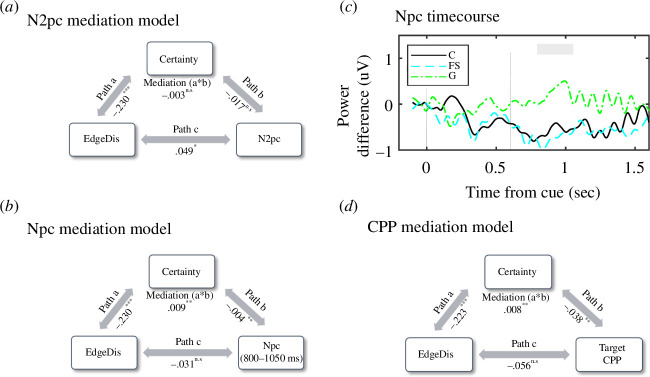
Mediation analysis with EdgeDis as the independent variable and certainty as the mediating variable. Path a illustrates the relationship between the predictor (EdgeDis) and the mediator (certainty). Path b denotes the relationship between the mediator and the outcome (N2pc), while controlling for the predictor. Path c represents the direct (unmediated) relationship between the predictor and the outcome. (*a*) Mediation model with N2pc as the outcome variable (no significant mediation effect). (*b*) Mediation model with Npc (during the 800–1050 ms time period) as the outcome variable (significant mediation effect). (*c*) Npc time course following the presentation of cue distinguished by certainty with a significant mediation period highlighted (grey-filled rectangle). (*d*) Mediation model with response-locked CPP peak as the outcome variable (significant mediation effect). In (*a*), (*b*) and (*d*), slope estimates are reported below arrows and significance is denoted as *p < 0.05, **p < 0.01, ***p < 0.001.

The mediation model for N2pc is displayed in figure 3*a*. Although EdgeDis directly predicted N2pcCue and also predicted certainty (as reported in the previous sections), there was no mediating effect of certainty for the N2pcCue component (a × b; ACME = 0.003, CI [−0.002-0.010], *p* = 0.242), as illustrated in figure 3*a*.

Time-series analysis identified a later period (800–1050 ms) in which certainty both predicted Npc magnitude (F_1,2065_ = 9.979, *p *< 0.01; *b* = −0.044, s.e. = 0.014, CI [−0.071 to −0.017]) and acted as a mediating factor between cue EdgeDis and Npc magnitude (a × b; ACME = 0.009, CI [0.002–0.010], *p *< 0.001, clusterP: *p *< 0.001), as illustrated in figure 3*b*. There was no significant direct effect of EdgeDis on Npc during this time period (F_1,5750_ = 0.007, *p* = 0.934), suggesting that this mediation occurred indirectly through the level of certainty. figure 3*c* displays the evolution of Npc following the cue, revealing an SPCN for fairly sure and certain trials, which is absent in guess trials.

### (d) Edge disruption and subjective rating as a driver of central parietal positivity (CPP)

We investigated whether response-locked CPP peak, serving as a signature of decision-making, was modulated by EdgeDis and TexRot. As there was no significant effect of cue validity on CPP (F_1,5170_ = 0.015, *p* = 0.902), all trials (cue valid and invalid) were included in the model. There was no indication that the physical properties of the initial cue stimulus significantly predicted response-locked CPP (EdgeDis: F_2,5170_ = 2.876, *p* = 0.09; TexRot: F_1, 5170_ = 0.279, *p* = 0.757; interaction: F_2,5170_ = 1.197, *p* = 0.302).

**Figure 4.**
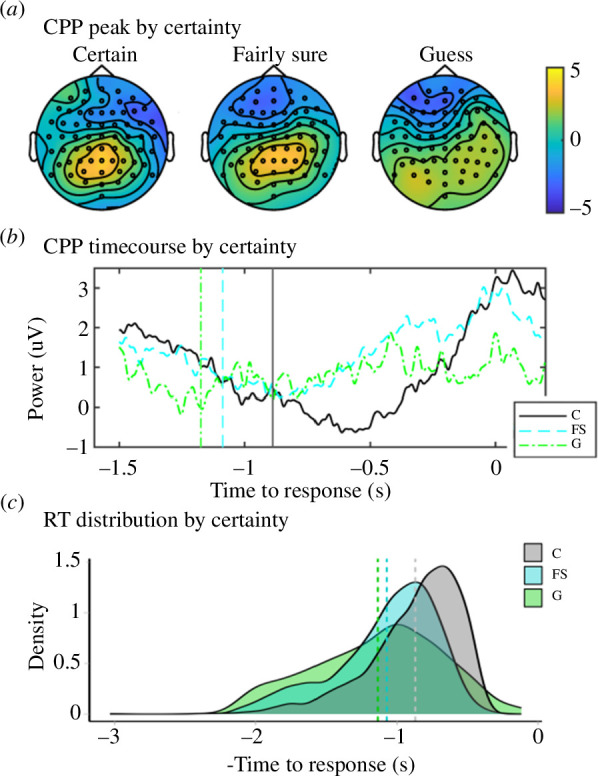
Central parietal positivity (CPP) neural signatures by certainty. (*a*) Topographic plots of CPP at the point of response (±100 ms) distinguished by certainty rating. (*b*) Response-locked central parietal ERP signature up until the point of response, distinguished by certainty (C = certain, FS = fairly sure, G = guess). ERPs were baseline-corrected to the point of stimulus onset on a trial-by-trial basis. (*c*) Distributions of negative RTs for comparison to CPP distribution in (*b*). Vertical lines in (*b*) and (*c*) represent the mean RTs for each level of certainty.

We investigated whether participant self-reported certainty predicted the amplitude of the CPP. Given that CPP build can be influenced by RT [18,33] and accuracy [19], these were added to the model in addition to certainty. Both certainty and RT predicted CPP amplitude at the time of response, while accuracy did not (certainty: F_2,5172_ = 4.501, *p *< 0.05; RT: F_1, 5164_ = 6.086, *p *< 0.01; accuracy: F_2,5163_ = 0.681, *p* = 0.409). The CPP component was evident as a typical focal peak over Pz for certain and fairly sure responses but was not evident for guess responses (figure 4*a*,*b*). Estimates for certainty (fairly sure: *b* = 0.102, s.e. = 0.035, CI [0.033–0.171]; certain: *b* = 0.086, s.e. = 0.035, CI [0.018–0.155]) represent the lack of the typical cumulative build of CPP for guess trials and the more typical build in fairly sure and certain trials (figure 4*b*). Estimates for RT (*b* = −0.180, s.e. = 0.073, CI [−0.323 to −0.037]) reflect the increased peak amplitude of CPP as RTs get shorter.

There was no significant direct effect of cue EdgeDis on response CPP peak amplitude (as reported above). However, we investigated whether certainty mediated the relationship between stimulus properties and response-locked CPP peak. We found that certainty was a mediating factor between EdgeDis and CPP amplitude (a × b; ACME = 0.008, CI [−0.01–0.00], *p *< 0.002; see figure 3*d*). Finally, there was no clear cue stimulus locked CPP signature (see electronic supplementary material, S6 for further detail).

## 4. Conclusions

In this study, we have demonstrated a novel experimental approach that reveals the neurophysiological components of how a common form of camouflage deflects the attention of other animals. While it has been established that edge disruption impairs search performance and consequently improves animals’ chances of survival [4,7], our method allows us to go beyond behavioural responses to camouflaged cues to uncover the specific attentional mechanisms that underlie detection and recognition. Crucially, our unique EEG cuing paradigm provides the first evidence of the neural basis for camouflage efficacy.

We found that the degree of edge disruption predicts both reaction times and accuracy rates in a behavioural decision-making experiment, wherein participants were tasked with detecting and delineating a camouflaged cue. From a behavioural standpoint, and novel to this study, we have uniquely shown that edge disruption can also influence self-reported subjective decision certainty. This is a factor that, in itself, is likely to influence performance, both in experimental search paradigms and in ecological settings. The rotation of the dominant orientation of the texture features interacted with edge disruption when predicting reaction times and certainty, but not accuracy. This suggests that deviation from the dominant texture orientation, along with edge disruption, makes observers more likely to notice the cue. However, it has less impact on accurately identifying the edges of the cue compared with edge disruption alone.

Our investigation of the propensity of edge disruption to modulate attention focus centred on N2pc. The N2pc is considered to signify the suppression of non-relevant information in the visual scene and the selection of task-relevant features [14], as well as the process of converting sensory input into a coherent neural representation during object identification [9,15]. The N2pc was consistently observed across different levels of EdgeDis following a camouflaged cue. However, its amplitude decreased with increasing EdgeDis. This, combined with the behavioural results, suggests that object edge disruption influences attentional reallocation in an experimental paradigm designed to simulate visual search in a natural scene. The modulation of the N2pc response may be attributable to the suppression of distracting information [36], such as the three non-cue panels and the surrounding background bark in the cue panel. However, since these distractors were luminance-matched across panels and, therefore, consistent across all EdgeDis values, it is more plausible to interpret variations in the N2pc as reflecting the selection of task-relevant features (specifically, detecting and extracting the edge and line orientation in the outline edge of the cue) and the conversion of these features into a coherent sensory representation of the object [9,15]. This process appears to be attenuated in cue stimuli with higher levels of edge disruption. Further evidence for this interpretation is that the N2pc following the cue did not vary with TexRot which, in itself, did not influence accuracy. Therefore, while increased texture discontinuation (perhaps representing a reduction in background matching) might lead to greater attentional focus on the cue and higher subjective certainty, the N2pc response suggests that accurate identification of the outline shape of the cue is influenced only by edge disruption. This notion might explain findings suggesting that disruptive coloration can effectively protect prey from predators, even when the prey does not necessarily blend with the background [37].

The N2pc response to the cue changed gradually with increasing edge disruption, indicating that this early stage of processing does not merely reflect a binary ‘detect’ or ‘not detect’ stage, but rather a stage where the visual representation of the cue progressively gains strength. Scaled variations in N2pc have been previously observed in relation to task difficulty or the number of objects to be delineated [38,39]. Thus, anomalies in the cue’s outline, with regard to line and edge orientation, slow the process of building an object representation. Supporting this interpretation, neural signatures in the occipital cortex exhibited a more obvious binary divergence only after the offset of the camouflaged cue. This manifested as an SCPN that was directly influenced by subjective certainty and indirectly influenced by EdgeDis through the mediating factor of subjective certainty. Specifically, there was a sustained period of occipital negativity contralateral to the cue location in fairly sure and certain trials that was completely absent in guess trials. This result probably reflects the sustained focus of attention on the side of the screen where the cue appears in trials in which participants were more confident [16,17]. Additionally, due to the task’s requirement to briefly hold the shape of the cue in memory to permit matching to the target, it is also probable that this result reflects the maintenance of the cue representation [16,17].

A binary divergence in neural signatures was also apparent in the response-locked CPP. Reflecting the SCPN result, CPP was directly predicted by subjective certainty and by EdgeDis only through the mediating factor of subjective certainty. The lack of distinct CPP following the cue, but a clear response-locked CPP following the target (when a match response was required), gives more weight to the interpretation that CPP was driven by subjective certainty or ‘conscious experience’ in the lead up to a decision, as opposed to evidence accumulation based purely on the physical stimulus properties [19]. This suggests a nuanced relationship between the accumulation of physical evidence and decision-making, probably influenced by the balance between signal (i.e. physical stimulus properties) and inherent noise within the neural network. It is conceivable that edge disruption, as a form of camouflage, exploits these neural intricacies to enhance the survival of a species. In the natural environment, decision confidence based on evidence accumulation is likely to influence the speed at which an attack is initiated. Meta-analysis has found that disruptive coloration increases search times by 57%, allowing prey escape if the prey are able to detect the predator [40]. However, this hypothesis requires further empirical testing with paradigms designed to directly influence decision confidence independently of camouflaged patterning.

The pattern of findings in this study is somewhat comparable to predictions of the ‘global neuronal workspace model’ purported by Dehaene and colleagues with regard to subliminal visual processing in attentional blink and masking paradigms [41,42]. While such paradigms were not utilized in the current study, the same principles could be applied easily. Activations in higher order visual areas exhibit a linear relationship with cue clarity (i.e. edge disruption), but a certain threshold must be surpassed before a ‘global ignition’ occurs. This leads to the activation of higher order parietal and prefrontal areas. Subsequently, reciprocal top-down and bottom-up connections propagate stimulus representation within a hierarchical network, resulting in a conscious ‘seen’ experience. This framework could readily explain findings from mock field studies using eye-tracking, where human participants passed over camouflaged prey during visual search without detecting them and displayed longer visual inspection times before attacking [4]. It is plausible that camouflaged patterning has evolved to exploit the neural architecture of potential predators, potentially by delaying or even preventing the global ignition process crucial for conscious detection and recognition.

To our knowledge, this study is the first to provide evidence supporting a plausible neural explanation for the delayed ‘attack’ response towards prey exhibiting high levels of edge disruption. These results also offer further validation that neurophysiologically derived computational metrics of edge disruption [7] correspond to human behavioural performance and thus serve as a reliable measure for quantifying camouflage. We propose that integrating psychological and neurophysiological approaches into ecological research offers a unique and crucial perspective for understanding how camouflage features exploit the observer’s neural architecture. Furthermore, we advocate for future studies to prioritize the consideration of contextual learning experiences to attain a more nuanced understanding of foraging behaviour in ecological contexts.

## Data Availability

All data and associated files are available via Dryad [43]. Supplementary materials are available online [44].
